# Financial toxicity profiles and associated factors among lung cancer patients receiving immunotherapy: a latent profile analysis

**DOI:** 10.3389/fpubh.2026.1909233

**Published:** 2026-10-16

**Authors:** Jing Nie, Peijuan Hu, Wei Qian, Xiaoyan Lu, Yulong Tan

**Affiliations:** Department of Thoracic Surgery, Huashan Hospital, Fudan University, Shanghai, China

**Keywords:** financial toxicity, immunotherapy, latent profile analysis, lung neoplasms, psychological resilience, social support

## Abstract

**Objectives:**

To identify latent profiles of patient-reported financial toxicity and factors associated with profile membership among lung cancer patients receiving immunotherapy.

**Methods:**

A total of 331 lung cancer patients receiving immunotherapy were recruited by convenience sampling. Financial toxicity, perceived social support, and psychological resilience were assessed using the Comprehensive Score for Financial Toxicity (COST-PROM), Perceived Social Support Scale (PSSS), and Connor-Davidson Resilience Scale (CD-RISC), respectively. Latent profile analysis (LPA) based on the 11 COST-PROM items was used to identify financial-toxicity profiles. Factors associated with profile membership were examined using the Mplus three-step R3STEP procedure, which accounts for classification uncertainty. A conventional multinomial logistic regression based on most-likely class assignment was used as a sensitivity analysis. Additional analyses examined the robustness of the LPA solution, an R3STEP model including all available covariates, collinearity, and possible nonlinear effects of PSSS and CD-RISC.

**Results:**

Three profiles were identified: Severe Financial Toxicity (28.7%), Moderate Financial Toxicity (39.9%), and Mild Financial Toxicity (31.4%). In R3STEP analyses, lower monthly income, out-of-pocket payment, and non-working status were associated with higher odds of Severe Financial Toxicity profile membership (*p* < 0.05), whereas higher PSSS and CD-RISC scores were associated with lower odds (*p* < 0.05). The modal-assignment sensitivity analysis yielded materially consistent findings. The three classes differed mainly in overall severity, with broadly parallel item profiles. Average posterior probabilities were 0.989, 0.982, and 0.992 for the Severe, Moderate, and Mild classes, respectively. Both alternative LPA models retained three classes, and the additional multivariable R3STEP model produced similar results.

**Conclusion:**

Patient-reported financial toxicity was heterogeneous among lung cancer patients receiving immunotherapy. Socioeconomic vulnerability and psychosocial characteristics were associated with profile membership. Routine financial-distress screening and financial navigation may be considered for patients with greater financial toxicity, while psychosocial intervention strategies require prospective evaluation before their effectiveness can be inferred. The classes mainly reflected different levels of financial toxicity rather than distinct response patterns.

## 1 Introduction

The incidence and mortality of primary lung cancer remain among the highest of all malignancies worldwide. According to GLOBOCAN 2022, approximately 2.48 million new lung cancer cases and 1.8 million lung cancer deaths occurred globally, accounting for 18.7% of all cancer deaths and making lung cancer the leading cause of cancer-related mortality (1). In recent years, immune checkpoint inhibitors, including PD-1/PD-L1 inhibitors, have improved outcomes for patients with lung cancer (2). However, prolonged treatment and combination regimens can impose a substantial financial burden. Out-of-pocket expenditure and insurance coverage are important components of cancer-related financial toxicity, including among patients with cancer in China (3–5).

Financial toxicity (FT) is a multidimensional patient-reported consequence of cancer-related costs, encompassing material hardship, psychological distress, and cost-related coping behaviors (6). Importantly, objective financial burden (e.g., out-of-pocket expenditures, income loss, debt, or depleted savings) and subjective financial toxicity are related but not interchangeable. COST-PROM primarily reflects patients’ perceived financial well-being and distress rather than directly measuring actual income, assets, or treatment expenditures. Prior studies have linked greater FT with poorer quality of life and cost-related coping behaviors (4, 7).

Many cross-sectional studies summarize FT using total or mean scale scores, implicitly treating patients as a homogeneous group. However, patient-reported financial distress may vary substantially across individuals. Latent profile analysis (LPA) provides a person-centered approach for identifying subgroups with distinct response patterns across observed indicators (8), and prior studies have applied LPA to characterize heterogeneity in financial toxicity among patients with cancer, including those with lung cancer (8–10). LPA can identify groups that differ mainly in severity as well as groups with distinct response patterns. Unlike categorizing patients using predefined cut-offs on the total score, LPA derives classes from the observed item responses while accounting for uncertainty in class membership.

FT may also be associated with psychosocial resources such as perceived social support and psychological resilience. Previous studies have reported associations among socioeconomic status, social support, self-efficacy, and FT (11, 12), as well as relationships between social support and psychological outcomes in patients with cancer (13, 14). These findings suggest that psychosocial resources may be relevant correlates of patient-reported financial distress. However, because much of the available evidence is cross-sectional, it cannot establish a buffering or protective causal mechanism.

Evidence remains limited regarding latent profiles of patient-reported FT specifically among lung cancer patients receiving immunotherapy and the socioeconomic and psychosocial characteristics associated with those profiles. Therefore, this study aimed to identify latent FT profiles using COST-PROM item responses and to examine factors associated with profile membership, including personal monthly income, payment method, employment status, perceived social support, and psychological resilience. A person-centered description of this heterogeneity may inform risk-stratified screening and the development of supportive-care and financial-navigation strategies that require prospective evaluation (15–17). Given the cross-sectional design, these analyses describe associations rather than causal relationships.

## 2 Methods

### 2.1 Study subjects

Using convenience sampling, we recruited lung cancer patients receiving immunotherapy in the Department of Thoracic Surgery at Huashan Hospital, Fudan University, between June and December 2025.

Inclusion criteria were: (1) pathologically or cytologically confirmed primary lung cancer; (2) current receipt of immunotherapy (e.g., PD-1/PD-L1 inhibitors); (3) age ≥ 18 years; (4) basic communication and reading comprehension ability; and (5) provision of informed consent and voluntary participation in the study.

Exclusion criteria were: (1) severe dysfunction of major organs such as the heart, liver, or kidneys; (2) a history of psychiatric disorders or cognitive impairment; and (3) extreme physical weakness or terminal-stage disease that precluded completion of the questionnaire.

A total of 350 questionnaires were distributed. After questionnaires with patterned responses or > 10% missing data were excluded, 331 valid questionnaires remained, yielding a valid response rate of 94.6%.

After on-site checking and exclusion of invalid questionnaires, the 331 retained questionnaires had no remaining item-level or covariate-level missing values; therefore, no imputation was required.

### 2.2 Instruments

#### 2.2.1 General information questionnaire

A general information questionnaire was developed by the researchers based on a literature review. It collected data on gender, age group, educational level, current employment status, primary caregiver, personal monthly income, number of comorbid chronic diseases, time since lung cancer diagnosis, duration of immunotherapy, and the primary payment method for current immunotherapy drugs.

#### 2.2.2 Comprehensive score for financial toxicity (COST-PROM)

This scale was developed by De Souza et al. (18) and translated and revised into a Chinese version by Yu et al. (19) in 2017. The scale contains 11 items and uses a 5-point Likert response format (0–4), with a total score ranging from 0 to 44; lower scores indicate greater patient-reported financial toxicity. COST-PROM is a subjective patient-reported measure and should not be interpreted as a direct measure of actual household wealth, savings, debt, or out-of-pocket expenditure. In this study, the Cronbach’s alpha coefficient was 0.831. Items 2, 3, 4, 5, 8, 9, and 10 were reverse-scored before analysis according to the COST scoring rules, so that higher scores consistently indicated better financial well-being and lower financial toxicity.

#### 2.2.3 Perceived social support scale (PSSS)

The PSSS, developed by Zimet et al. (20) and revised into a Chinese version by Wang et al. (21), was adopted. The scale contains 12 items, covering three dimensions: family support, friend support, and other support, with 4 items in each dimension. A 7-point Likert scale (1–7 points) is used, and the total score ranges from 12 to 84. A higher score indicates a higher level of perceived social support. In this study, Cronbach’s α was 0.895.

#### 2.2.4 Connor-Davidson resilience scale (CD-RISC)

The Chinese version of the CD-RISC-25, developed by Connor and Davidson (22) and localized by Yu et al. (23), was used. The scale contains 25 items across three dimensions: tenacity, strength, and optimism. It uses a 5-point Likert scale (0–4), with a total score ranging from 0 to 100; higher scores indicate greater psychological resilience. In this study, Cronbach’s α was 0.912.

### 2.3 Data collection

After obtaining approval from the department director and head nurse, trained researchers used standardized instructions to explain the study purpose and procedures to eligible patients. After written informed consent was obtained, participants completed the questionnaire on site. For participants unable to complete the questionnaire independently because of impaired vision or limited literacy, a researcher read the items aloud and recorded the participant’s responses objectively and neutrally. Questionnaires were checked immediately after completion, and participants were asked to complete any omitted items when appropriate.

### 2.4 Data analysis

Data analysis was performed using SPSS 26.0 and Mplus 8.3. LPA was conducted using the 11 COST-PROM item scores (0–4), which were treated as approximately continuous indicators. The continuous-indicator mixture model assumes approximate conditional normality of the observed indicators within each latent class. For all one- through five-class solutions, item means were freely estimated across classes, residual variances were constrained to be equal across classes, and within-class residual covariances were fixed to zero, consistent with local independence. This equal residual-variance/zero residual-covariance specification was intentionally retained across all class-enumeration models to maintain a common, parsimonious measurement structure and direct comparability among candidate solutions; alternative residual variance/covariance parameterizations were not introduced during class enumeration. Models were estimated by maximum likelihood with robust standard errors. One- through five-class models were compared using the Akaike Information Criterion (AIC), Bayesian Information Criterion (BIC), sample-size-adjusted BIC (aBIC), entropy, the Lo–Mendell–Rubin adjusted likelihood ratio test (LMRT), and the bootstrap likelihood ratio test (BLRT). Model selection was based on the combined evidence from information criteria, likelihood-ratio tests, entropy, class size, parsimony, and clinical interpretability rather than on any single fit statistic. Two additional sensitivity analyses were conducted: a continuous-indicator LPA allowing residual variances to vary across classes and an ordinal LPA treating the 0–4 COST-PROM items as ordinal indicators. Model convergence, entropy, class proportions, and profile patterns were compared with the primary model.

Descriptive analyses were conducted in SPSS 26.0. Normally distributed continuous variables were summarized as mean ± standard deviation and compared using one-way ANOVA; categorical variables were summarized as frequencies and percentages and compared using Pearson’s chi-square tests. Candidate covariates with *p* < 0.10 in univariate analyses were entered into the association model. To account for uncertainty in latent-profile classification, associations between covariates and profile membership were estimated in Mplus 8.3 using the R3STEP auxiliary-variable procedure, with Mild Financial Toxicity as the reference latent class. Reference categories were monthly income > 8,000 CNY, medical insurance reimbursement, and employed status. R3STEP preserves the measurement model from the selected LPA solution while adjusting the multinomial regression for classification error. As a sensitivity analysis, a conventional multinomial logistic regression based on most-likely (modal) class assignment was performed in SPSS. All tests were two-sided, and *p* < 0.05 was considered statistically significant.

As an additional analysis, an R3STEP model included age, gender, educational level, number of comorbid chronic diseases, time since diagnosis, duration of immunotherapy, employment status, monthly income, payment method, PSSS, and CD-RISC regardless of univariate *p* values. Collinearity between PSSS and CD-RISC was assessed using Pearson’s correlation, variance inflation factors (VIFs), and tolerance values. Quadratic terms were added to examine possible nonlinearity.

### 2.5 Ethical considerations

This study was conducted in accordance with the Declaration of Helsinki and was approved by the Ethics Committee of Huashan Hospital, Fudan University (Approval Code: 20241358) before data collection. Before completing the questionnaire, all eligible patients received a detailed explanation of the study purpose, procedures, and potential risks and benefits. Participation was entirely voluntary, and patients were informed that they could withdraw at any time without affecting subsequent immunotherapy or nursing care. All data were anonymized and used solely for this research, and participant confidentiality was strictly protected. All participants provided written informed consent.

## 3 Results

### 3.1 Latent profile analysis of financial toxicity in lung cancer patients undergoing immunotherapy

The 11 COST-PROM items from 331 participants were used as observed indicators, and models with one to five latent classes were estimated (Table 1).

**Table 1:** Model-fit indices for latent profile analysis of financial toxicity among lung cancer patients receiving immunotherapy (*N* = 331).

Model	AIC	BIC	aBIC	LMRT P	BLRT P	Entropy	Class proportions (%)
1-Class	11639.241	11722.888	11653.103				
2-Class	10211.768	10341.040	10233.191	<0.001	<0.001	0.946	31.7; 68.3
3-Class	9707.093	9881.991	9736.078	<0.001	<0.001	0.950	28.7; 39.9; 31.4
4-Class	9700.163	9920.686	9736.708	0.753	0.146	0.874	27.5; 11.8; 26.2; 34.5
5-Class	9696.024	9962.172	9740.130	0.395	0.667	0.871	6.3; 27.8; 11.2; 23.3; 31.4

The three-class model had AIC = 9707.093, BIC = 9881.991, aBIC = 9736.078, entropy = 0.950, and significant LMRT and BLRT results (both *p* < 0.001). The four-class model had a slightly lower AIC (9700.163, difference = −6.930), but its BIC increased to 9920.686, its aBIC increased slightly to 9736.708, and its entropy decreased to 0.874. Neither the LMRT (*p* = 0.753) nor the BLRT (*p* = 0.146) supported improvement over the three-class model. The five-class model was also unsupported by the LMRT (*p* = 0.395) and BLRT (*p* = 0.667) and contained a small class representing 6.3% of the sample. Therefore, considering statistical fit, class separation, class size, parsimony, and interpretability together, the three-class solution was retained.

The class-specific average posterior probabilities were 0.989 for Severe Financial Toxicity, 0.982 for Moderate Financial Toxicity, and 0.992 for Mild Financial Toxicity. In the continuous-indicator sensitivity model allowing class-specific residual variances, three classes were retained, with proportions of 29.0%, 39.6%, and 31.4% and entropy = 0.950. The ordinal LPA also retained three classes, with proportions of 29.3%, 40.5%, and 30.2% and entropy = 0.940. These results were similar to those of the primary model.

The profile patterns are shown in Figure 1. Class 1 (Severe Financial Toxicity) included 95 participants (28.7%) and had the lowest COST-PROM item scores, indicating the greatest patient-reported financial distress. Class 2 (Moderate Financial Toxicity) included 132 participants (39.9%) and had intermediate item scores. Class 3 (Mild Financial Toxicity) included 104 participants (31.4%) and had the highest item scores, indicating comparatively less patient-reported financial distress. These labels describe relative COST-PROM severity and do not imply direct measurement of objective financial resources. The three curves were broadly parallel across the 11 items, indicating that the classes differed mainly in overall severity rather than in item-specific response patterns.

**Figure 1:**
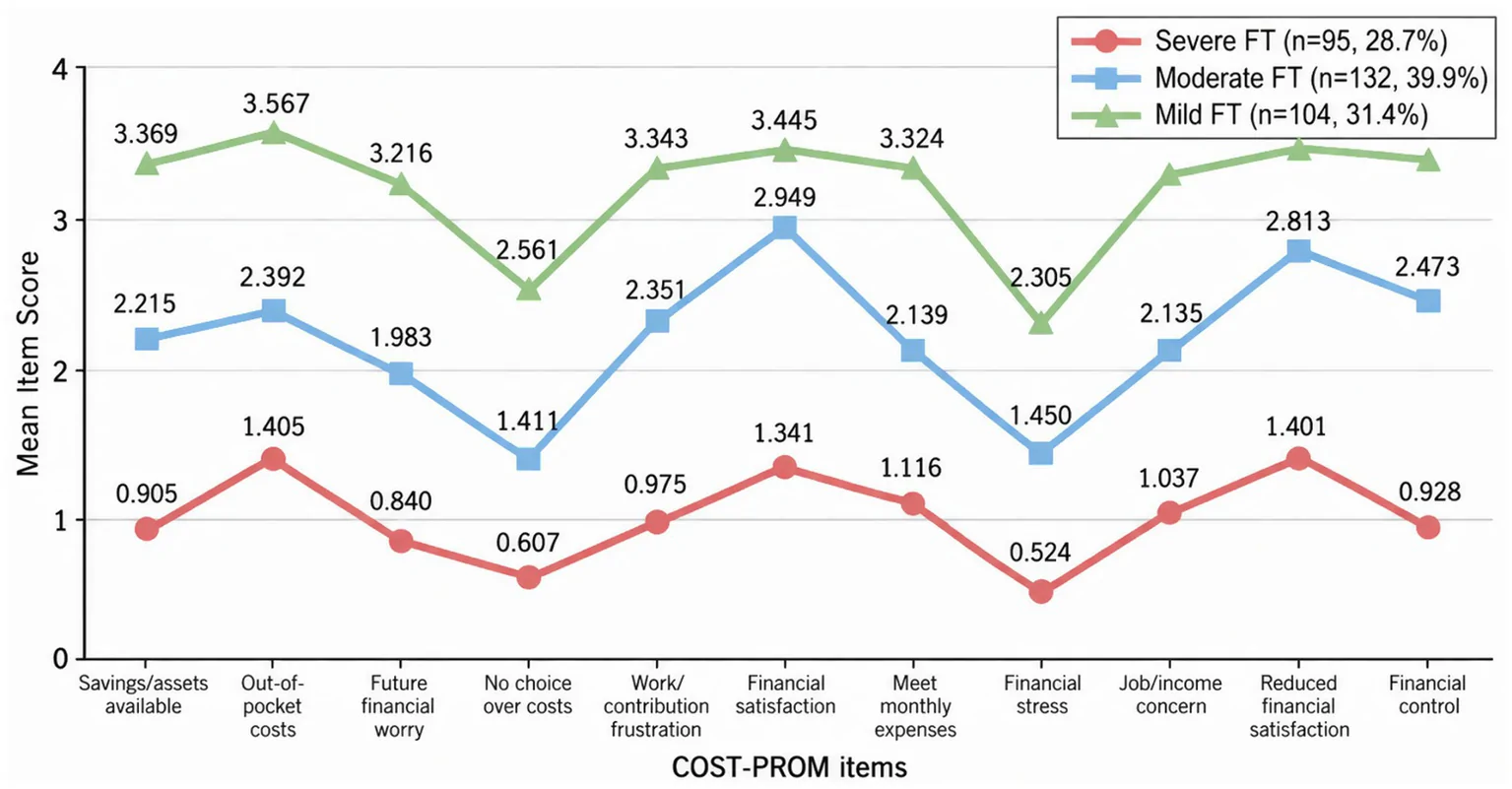
Latent profiles of financial toxicity among lung cancer patients receiving immunotherapy. (Items requiring reverse scoring were recoded before LPA; higher scores indicate better financial well-being and lower financial toxicity).

COST-PROM total scores were 11.1 ± 3.3 in the Severe Financial Toxicity class, 24.3 ± 3.5 in the Moderate class, and 35.3 ± 3.1 in the Mild class (one-way ANOVA, *p* < 0.001).

### 3.2 Comparison of general data and psychosocial characteristics among patients in different latent profiles of financial toxicity

Univariate analyses showed significant differences among the three profiles in employment status, personal monthly income, payment method, PSSS total score, and CD-RISC total score (all *p* < 0.001). Age, gender, educational level, primary caregiver, number of comorbid chronic diseases, time since diagnosis, and duration of immunotherapy were not significantly different across profiles (all *p* > 0.05) and did not meet the prespecified *p* < 0.10 screening criterion for entry into the multinomial model (Table 2). These variables were nevertheless included in the additional multivariable sensitivity model.

**Table 2:** Comparison of demographic, clinical, and psychosocial characteristics across latent financial-toxicity profiles (*N* = 331).

Variables	Class 1: Severe FT (*n* = 95)	Class 2: Moderate FT (*n* = 132)	Class 3: Mild FT (*n* = 104)	χ^2^/F	*P*
Age [*n* (%)]				4.504ᵃ	0.342
< 60 years	38 (32.8%)	43 (37.1%)	35 (30.2%)		
60–69 years	36 (25.2%)	65 (45.5%)	42 (29.4%)		
≥ 70 years	21 (29.2%)	24 (33.3%)	27 (37.5%)		
Gender [*n* (%)]				0.432ᵃ	0.806
Male	53 (27.7%)	79 (41.4%)	59 (30.9%)		
Female	42 (30.0%)	53 (37.9%)	45 (32.1%)		
Educational level [*n* (%)]				3.467ᵃ	0.483
Middle school and below	23 (27.7%)	28 (33.7%)	32 (38.6%)		
High school or equivalent	43 (29.3%)	59 (40.1%)	45 (30.6%)		
College and above	29 (28.7%)	45 (44.6%)	27 (26.7%)		
Employment status [*n* (%)]				29.000ᵃ	<0.001
Employed	22 (20.8%)	35 (33.0%)	49 (46.2%)		
Retired	29 (23.6%)	64 (52.0%)	30 (24.4%)		
Sick leave/unemployed	44 (43.1%)	33 (32.4%)	25 (24.5%)		
Primary caregiver [n (%)]				7.120ᵃ	0.310
Spouse	66 (34.0%)	70 (36.1%)	58 (29.9%)		
Children	21 (20.0%)	49 (46.7%)	35 (33.3%)		
Other relatives/friends	5 (25.0%)	8 (40.0%)	7 (35.0%)		
Care worker/Nanny	3 (25.0%)	5 (41.7%)	4 (33.3%)		
Monthly income, CNY [*n* (%)]				58.671ᵃ	<0.001
< 3000	40 (48.8%)	31 (37.8%)	11 (13.4%)		
3000–5000	30 (35.3%)	42 (49.4%)	13 (15.3%)		
5001–8000	13 (12.5%)	41 (39.4%)	50 (48.1%)		
> 8000	12 (20.0%)	18 (30.0%)	30 (50.0%)		
Number of Comorbidities [*n* (%)]				1.299ᵃ	0.861
0	38 (28.8%)	52 (39.4%)	42 (31.8%)		
1	39 (30.0%)	54 (41.5%)	37 (28.5%)		
≥ 2	18 (26.1%)	26 (37.7%)	25 (36.2%)		
Time since diagnosis [*n* (%)]				10.123ᵃ	0.120
< 1 year	33 (37.1%)	34 (38.2%)	22 (24.7%)		
1–3 years	37 (27.6%)	47 (35.1%)	50 (37.3%)		
3–5 years	15 (19.7%)	38 (50.0%)	23 (30.3%)		
> 5 years	10 (31.3%)	13 (40.6%)	9 (28.1%)		
Duration of immunotherapy [*n* (%)]				7.898ᵃ	0.246
< 6 months	29 (36.3%)	32 (40.0%)	19 (23.8%)		
6–12 months	25 (24.0%)	47 (45.2%)	32 (30.8%)		
13–24 months	20 (23.8%)	33 (39.3%)	31 (36.9%)		
> 24 months	21 (33.3%)	20 (31.7%)	22 (34.9%)		
Payment method [*n* (%)]				42.451ᵃ	<0.001
Medical insurance reimbursement	34 (17.9%)	73 (38.4%)	83 (43.7%)		
Out-of-pocket payment	50 (46.7%)	42 (39.3%)	15 (14.0%)		
Charity drug donation/free trials	11 (32.4%)	17 (50.0%)	6 (17.6%)		
PSSS total score (Mean ± SD)	55.40 ± 13.05	62.55 ± 10.99	67.06 ± 9.49	27.262ᵇ	<0.001
CD-RISC total score (Mean ± SD)	55.36 ± 14.52	61.48 ± 12.26	68.28 ± 11.46	25.729ᵇ	<0.001

^a^chi-square test; ^b^one-way ANOVA. Percentages for categorical variables are row percentages.

### 3.3 Multinomial logistic regression analysis of the latent profiles of financial toxicity in lung cancer patients undergoing immunotherapy

Associations between the five covariates that met the prespecified univariate screening criterion and latent-profile membership were estimated using the Mplus R3STEP procedure, with Mild Financial Toxicity as the reference profile (Table 3). This three-step approach accounts for classification uncertainty. A conventional multinomial logistic regression based on modal class assignment was also performed as a sensitivity analysis. The additional multivariable R3STEP analysis produced similar results. PSSS and CD-RISC were moderately correlated (*r* = 0.52, *p* < 0.001). VIFs were 1.4–1.5 and tolerance values were 0.68–0.75, with no indication of problematic multicollinearity. Quadratic terms were not significant for PSSS (*p* = 0.34) or CD-RISC (*p* = 0.41).

**Table 3:** Three-step multinomial logistic regression (R3STEP) of factors associated with latent financial toxicity profile membership (*N* = 331).

**Variable**	**Severe Financial Toxicity vs Mild Financial Toxicity**					**Moderate Financial Toxicity vs Mild Financial Toxicity**				
	**B**	**SE**	**OR**	**95% CI**	**P**	**B**	**SE**	**OR**	**95% CI**	**P**
Income <3000 CNY	2.680	0.640	14.585	4.160–51.131	<0.001	1.770	0.540	5.871	2.037–16.918	0.001
Income 3000–5000 CNY	1.980	0.610	7.243	2.191–23.941	0.001	1.810	0.500	6.110	2.293–16.281	<0.001
Income 5001–8000 CNY	0.270	0.580	1.310	0.420–4.083	0.642	0.540	0.430	1.716	0.739–3.986	0.209
Out-of-pocket payment	2.570	0.490	13.066	5.001–34.138	<0.001	1.450	0.410	4.263	1.909–9.522	<0.001
Charity/free trial	1.490	0.660	4.437	1.217–16.177	0.024	0.940	0.560	2.560	0.854–7.672	0.093
Retired	1.080	0.490	2.945	1.127–7.694	0.028	1.240	0.380	3.456	1.641–7.278	0.001
Sick leave/unemployed	1.610	0.500	5.003	1.878–13.330	0.001	0.700	0.410	2.014	0.902–4.498	0.088
PSSS	-0.089	0.019	0.915	0.881–0.950	<0.001	-0.036	0.016	0.965	0.935–0.995	0.024
CD-RISC	-0.097	0.017	0.908	0.878–0.938	<0.001	-0.051	0.014	0.950	0.925–0.977	<0.001

Mild Financial Toxicity was the reference profile.R3STEP accounts for classification uncertainty; estimates for sparse payment-method categories should be interpreted cautiously. Reference categories were monthly income > 8000 CNY, medical insurance reimbursement, and employed status. OR, odds ratio; CI, confidence interval; PSSS, Perceived Social Support Scale; CD-RISC, Connor-Davidson Resilience Scale. Associations were estimated using the Mplus R3STEP procedure to account for latent-class classification uncertainty. A conventional modal-assignment multinomial logistic regression was used as a sensitivity analysis and produced similar results. For clinical interpretation, the ORs per 10-point increase were: Severe vs Mild, PSSS 0.411 (95% CI 0.282–0.599) and CD-RISC 0.381 (95% CI 0.272–0.527); Moderate vs Mild, PSSS 0.700 (95% CI 0.511–0.951) and CD-RISC 0.599 (95% CI 0.459–0.792).

For Severe versus Mild Financial Toxicity, monthly income < 3,000 CNY (OR = 14.585, 95% CI 4.160–51.131, *p* < 0.001) and 3,000–5,000 CNY (OR = 7.243, 95% CI 2.191–23.941, *p* = 0.001) were associated with higher odds of membership in the Severe profile. Income of 5,001–8,000 CNY was not statistically significant (OR = 1.310, 95% CI 0.420–4.083, *p* = 0.642). Out-of-pocket payment (OR = 13.066, 95% CI 5.001–34.138, *p* < 0.001), charity drug donation/free trials (OR = 4.437, 95% CI 1.217–16.177, *p* = 0.024), retirement (OR = 2.945, 95% CI 1.127–7.694, *p* = 0.028), and sick leave/unemployment (OR = 5.003, 95% CI 1.878–13.330, *p* = 0.001) were also associated with higher odds of membership in the Severe profile. Higher PSSS scores (OR = 0.915 per point, 95% CI 0.881–0.950, *p* < 0.001) and CD-RISC scores (OR = 0.908 per point, 95% CI 0.878–0.938, *p* < 0.001) were associated with lower odds of Severe-profile membership. For clinical interpretability, the corresponding ORs per 10-point increase were 0.411 for PSSS (95% CI 0.282–0.599) and 0.381 for CD-RISC (95% CI 0.272–0.527).

For Moderate versus Mild Financial Toxicity, income < 3,000 CNY (OR = 5.871, 95% CI 2.037–16.918, *p* = 0.001), income 3,000–5,000 CNY (OR = 6.110, 95% CI 2.293–16.281, *p* < 0.001), out-of-pocket payment (OR = 4.263, 95% CI 1.909–9.522, *p* < 0.001), and retirement (OR = 3.456, 95% CI 1.641–7.278, *p* = 0.001) were associated with higher odds of membership in the Moderate profile. Higher PSSS scores (OR = 0.965, 95% CI 0.935–0.995, *p* = 0.024) and CD-RISC scores (OR = 0.950, 95% CI 0.925–0.977, *p* < 0.001) were associated with lower odds. Income of 5,001–8,000 CNY, charity drug donation/free trials, and sick leave/unemployment were not statistically significant for Moderate versus Mild Financial Toxicity (all *p* > 0.05). The modal-assignment sensitivity analysis yielded materially similar effect directions and the same pattern of statistical significance as the R3STEP analysis. Estimates for the charity drug donation/free-trial category remained comparatively imprecise because this subgroup was small (n = 34 overall: 11 Severe, 17 Moderate, and 6 Mild), as reflected by the wide confidence intervals. On the same 10-point scale, the ORs were 0.700 for PSSS (95% CI 0.511–0.951) and 0.599 for CD-RISC (95% CI 0.459–0.792).

## 4 Discussion

### 4.1 Heterogeneity of financial toxicity in lung cancer patients undergoing immunotherapy

LPA identified three profiles that differed in the relative severity of patient-reported FT. The Severe Financial Toxicity profile accounted for 28.7% of participants and had the lowest COST-PROM item scores. These scores indicate greater perceived financial distress, but they do not directly demonstrate the amount of out-of-pocket expenditure, household wealth depletion, treatment nonadherence, or quality-of-life impairment in this sample. Previous studies have linked greater FT with psychological distress and poorer patient-reported outcomes, including quality of life (24, 25). Accordingly, this subgroup may be particularly relevant for early financial-distress screening and referral to financial-navigation or social-work resources. The three curves were broadly parallel, suggesting that the classes mainly reflected differences in severity rather than distinct item-response patterns. LPA nevertheless provided a data-driven classification while accounting for uncertainty in class assignment. The separation in COST-PROM total scores and the similar results from the two alternative LPA models supported the stability of the three-class solution.

The Moderate Financial Toxicity profile accounted for 39.9% of participants and showed intermediate COST-PROM item scores. This neutral, data-driven label was used because the LPA itself does not demonstrate a compensatory psychosocial mechanism. Although psychosocial characteristics differed across profiles, the cross-sectional design cannot establish that social support or resilience caused a buffering effect. Supportive approaches such as counseling, peer/family support, or resilience-oriented care may be considered as hypotheses for prospective evaluation rather than interventions proven effective by the present study. The Moderate class therefore appears to represent an intermediate level of financial toxicity rather than a distinct response pattern.

The Mild Financial Toxicity profile accounted for 31.4% of participants and had the highest COST-PROM item scores, indicating comparatively less subjective financial distress. This label describes relative severity of patient-reported FT and does not imply abundant objective financial resources because COST-PROM does not directly measure income, savings, assets, or insurance adequacy. Longitudinal research in lung cancer has shown that FT can change substantially over time and that associated factors may differ across treatment phases (26). Therefore, even patients with relatively mild FT at one assessment may benefit from periodic reassessment during prolonged treatment. Because this was a single-center sample, the class proportions should be confirmed in other settings.

### 4.2 Factors associated with latent profiles of financial toxicity in lung cancer patients receiving immunotherapy

#### 4.2.1 Personal monthly income

Lower personal monthly income was associated with higher odds of membership in the Severe Financial Toxicity profile. Compared with patients reporting monthly income > 8,000 CNY, those reporting < 3,000 CNY or 3,000–5,000 CNY had higher odds of Severe rather than Mild Financial Toxicity profile membership, consistent with previous evidence linking lower income to greater financial hardship during cancer treatment (4, 27). Limited disposable income and savings may plausibly reduce a household’s capacity to absorb sustained treatment-related costs. However, the present cross-sectional study did not directly measure household assets, cumulative expenditure, or temporal pathways. Income may therefore be useful as one component of financial-distress screening rather than as a causal predictor. Financial counseling or navigation for lower-income patients should be evaluated prospectively.

#### 4.2.2 Payment method

Out-of-pocket payment was associated with higher odds of Severe Financial Toxicity profile membership than medical insurance reimbursement, consistent with evidence linking out-of-pocket expenditure and insurance coverage to FT (4, 5). Direct payment may expose patients more fully to treatment costs, although actual expenditure was not measured in the present study. From a health-economics perspective, insurance design, reimbursement eligibility, and access to assistance programs may shape the financial burden borne by patients. Clinical teams may consider early verification of reimbursement status and referral to financial-navigation resources, but the effectiveness of such strategies was not tested here.

#### 4.2.3 Current employment status

Non-working status (sick leave/unemployment or retirement) was associated with higher odds of Severe Financial Toxicity profile membership compared with employment. Loss or reduction of earned income during cancer treatment is a plausible pathway linking employment disruption with financial hardship, but the temporal direction cannot be established in this cross-sectional study. Employment status should therefore be interpreted as an associated socioeconomic marker rather than a causal risk factor.

#### 4.2.4 Perceived social support

Higher perceived social support was associated with lower odds of Severe Financial Toxicity profile membership. This finding is consistent with studies reporting relationships among social support, financial toxicity, and psychological outcomes in cancer populations (12, 14, 28, 29). Potential mechanisms may include access to informational, emotional, and practical resources; however, the present study did not test mediation, buffering, or intervention effects. Assessment of social-support needs and referral to peer, family, social-work, or community resources may be reasonable supportive-care options, but their effects on FT require prospective evaluation. Because social support and financial toxicity were measured at the same time, the direction of this association cannot be determined.

#### 4.2.5 Psychological resilience

Higher psychological resilience was associated with lower odds of Severe Financial Toxicity profile membership. Resilience may influence how patients appraise and cope with financial stress, but the cross-sectional association observed here cannot establish a protective causal effect (30). Resilience-enhancing interventions have improved resilience and some psychological outcomes in cancer populations (31), but the present study did not evaluate cognitive-behavioral therapy, narrative nursing, mindfulness, or any other intervention for FT. These approaches should therefore be considered potential strategies for prospective testing rather than effects demonstrated by this study. Because resilience and financial toxicity were measured at the same time, the direction of this association cannot be determined.

### 4.3 Public health and health-economics implications

Financial toxicity should not be framed solely as an individual coping problem. The observed associations with income, employment status, and payment method point to upstream determinants related to insurance protection, treatment affordability, employment disruption, and access to financial assistance. At the health-system level, standardized FT screening integrated into oncology pathways, followed by tiered referral to financial counselors, social workers, or navigation services, may help identify patients requiring support (16, 17). At the policy level, reimbursement design, out-of-pocket cost exposure, and access to assistance programs warrant consideration. These implications are hypothesis-generating because the current study did not evaluate the effectiveness or cost-effectiveness of specific clinical or policy interventions.

### 4.4 Limitations

This study has several limitations. First, its cross-sectional design precludes temporal or causal inference; financial toxicity, social support, and resilience were measured at the same time by self-report, so reverse causation and common-method effects cannot be excluded. Second, participants were recruited by convenience sampling from a single tertiary hospital, which limits generalizability to other institutions, regions, insurance systems, and immunotherapy settings. Third, COST-PROM reflects subjective financial distress and does not directly measure objective expenditures, household income dynamics, savings, debt, or catastrophic health spending. Fourth, several potentially important clinical and treatment-related variables—including cancer stage, histological subtype, specific immune-checkpoint inhibitor, monotherapy versus combination therapy, line of treatment, number of treatment cycles, objective treatment response, immune-related adverse events, cumulative treatment expenditure, and detailed reimbursement proportions beyond the primary payment method recorded in the questionnaire—were not systematically collected. Residual confounding by disease severity, treatment intensity, and reimbursement context therefore remains possible. Fifth, although the additional multivariable R3STEP analysis produced similar results, residual confounding cannot be excluded. The three-class solution was also similar in the alternative continuous and ordinal LPA models, but external validation is still needed. Sixth, some predictor subgroups, particularly the charity drug donation/free-trial category, were small, resulting in wide confidence intervals and potentially unstable estimates. Finally, financial toxicity was assessed at a single time point, so changes during immunotherapy could not be evaluated. Future studies should use multicenter prospective longitudinal designs, include objective economic measures and more detailed treatment variables, and replicate the class structure in independent samples.

## 5 Conclusion

This study identified three profiles of patient-reported financial toxicity among lung cancer patients receiving immunotherapy: Severe Financial Toxicity (28.7%), Moderate Financial Toxicity (39.9%), and Mild Financial Toxicity (31.4%). Lower monthly income, out-of-pocket payment, and non-working status were associated with higher odds of Severe Financial Toxicity profile membership, whereas higher perceived social support and psychological resilience were associated with lower odds. These findings support a person-centered approach to FT screening and highlight socioeconomic and psychosocial characteristics that may help identify patients requiring additional assessment. Financial-navigation and psychosocial-support strategies should be evaluated prospectively before conclusions are drawn about their effects on treatment adherence, quality of life, survivorship, or financial outcomes. Overall, the three classes mainly reflected different levels of financial toxicity rather than distinct response patterns. The class proportions should be confirmed in other populations and treatment settings. The three-class solution and the main covariate associations were similar in sensitivity analyses.

## Data Availability

The raw data supporting the conclusions of this article will be made available by the authors, without undue reservation.
